# Editorial: Creativity in Pathological Brain Conditions Across the Lifespan

**DOI:** 10.3389/fpsyg.2022.932399

**Published:** 2022-06-08

**Authors:** Barbara Colombo, Alice Cancer, Lindsey Carruthers, Alessandro Antonietti

**Affiliations:** ^1^Behavioral Neuroscience Lab, Champlain College, Burlington, VT, United States; ^2^Department of Psychology, Università Cattolica del Sacro Cuore, Milan, Italy; ^3^School of Applied Sciences, Edinburgh Napier University, Edinburgh, United Kingdom

**Keywords:** creativity, divergent thinking, neurodevelopmental disorders, brain damage, neurological conditions

Accumulated evidence showed that, under certain circumstances, creative thinking skills are enhanced in diverse neurodevelopmental disorders and pathological brain conditions. What are the mechanisms underlying the relationship between creativity and atypical brain functioning? On the one hand, it has been proposed that this relation is rooted in executive functions (Cancer et al., 2022). Divergent thinking benefits from an impairment of inhibitory processes, where a depletion of resources is often associated with atypical neural development and acquired brain impairments. Research also shows a crucial role of the dopaminergic system in creative thinking, with conditions characterized by dopamine imbalance supporting creative drive. Also, processing anomalies in diverse cognitive functions (e.g., hierarchical visual perception, working memory updating) and the preference for impulsive over deliberate behaviors (which are associated with several neurodevelopmental disorders), as well as the tendency to connect disparate notions (Cancer et al., 2016), have been hypothesized to enhance creative cognition. However, previous experimental investigations on clinical populations have reported inconsistent findings, due to the wide variety of tests and measures of creative performance, and thus it is not clear why atypical mind/brain functioning can sometimes favor creativity.

Some interesting and promising data have been reported in the literature. For example, a study on the genetic and neural basis of creativity (Liu et al., 2018) showed that high creativity appears to be associated with brain networks with strong top-down control vs. weak bottom-up processes and that genes correlated with creativity were also involved in glutamate and GABA functionality. Interestingly, some of these functions have been linked to specific clinical conditions. Top-down control network functioning has been linked to emotional instability associated with self-regulation in Tourette Syndrome (TS) and Obsessive-Compulsive Disorder (OCD) (Zhang et al., 2011) and with some of the motor symptoms of Parkinson's Disease (PD) (Chen et al., 2021). Moreover, the link between GABA and several major neurological disorders such as Huntington's chorea, PD, and Alzheimer's disease and other psychiatric disorders is also well known and used as basis for possible treatments (Gajcy et al., 2010; Lopatina et al., 2019). This line of research, which is quite promising from a theoretical standpoint, could also lead to implications relevant for clinical practice, by way of suggesting new approaches to build more motivating and effective interventions targeted to specific patient populations and by stressing the need to fuel cognitive reserve, which has been proved to be supported by creative jobs and hobbies, and to prevent cognitive decline in aging (Colombo et al., 2018; Fusi et al., 2020).

Based on this framework, this Research Topic aimed to further the knowledge on the cognitive and neural basis of creativity by studying clinical populations. More precisely, a collection of empirical research and theoretical contributions explored the hypothesis of enhanced creative thinking skills as a function of pathological conditions, namely, PD, Frontotemporal Dementia (FTD), TS, and Developmental Dyscalculia (DD).

Based on reported bursts of creativity following dopamine replacement therapy (DRT), Salvi et al. conducted an empirical study on PD patients. Divergent thinking and creative problem-solving performances of patients on medication were compared to those of a group of matched healthy controls. Contrary to the initial hypothesis, results did not provide strong evidence that DRT improved creative thinking in PD patients. Rather, PD was associated with less flexibility in divergent thinking and a lower performance in creative problem solving, compared to healthy controls. Authors suggested that reports of compulsive artistic production after DRT could be explained by worsened inhibitory control, which is detrimental for creative thinking, highlighting the need to overcome the “art bias”, according to which creativity matches artistic talent.

Starting from similar evidence of augmented “drive to produce” in patients with FTD, Fusi et al. reviewed a series of clinical studies to examine the extent to which divergent thinking was preserved in patients affected by FTD. Differential creative performances were associated with specific neurofunctional features of FTD. Specifically, the behavioral form of FTD was associated with the production of many ideas, through unimpaired access to memory stores. However, damages in the pre-frontal cortex impaired the ability to flexibly recombine the information to produce original ideas. Additionally, patients affected by the semantic variant of FTD showed fluency impairments, due to the degradation of their semantic memory store.

Although patients with TS often exhibit creative attitudes in artistic domains, only a few empirical studies explored the association between TS and creative thinking. In their theoretical contribution, Colautti et al. proposed a neurological hypothesis supporting TS association with creativity. Namely, as creative thinking is fostered by dopaminergic neurotransmission, the TS-associated hypersensitivity to postsynaptic dopamine receptors may facilitate creative processes. Furthermore, the authors reviewed the findings from studies showing higher mean scores of TS patients in divergent thinking tasks, comparison which, however, did not reach statistical significance, hence emphasizing the need for further research on this association.

Finally, Magenes et al. explored the hypothesis of a positive relationship between creativity and Developmental Dyscalculia (DD), after revealing a gap in the literature about this topic. A single empirical study previously reported lower creative performances of children with DD, compared to their typical peers. However, according to the authors, a possible link between creativity and DD could be supported by a neurofunctional overlap of neural structures involved in both DD and creative thinking, specifically enhanced activations of the angular gyrus (AG), the ventral medial pre-frontal cortex (VMPFC), and the inferior frontal gyrus (IFG). Furthermore, the cognitive peculiarities of students with DD, such as the use of unconventional strategies to solve computations, the preference for global information processing, and an attentional dysfunction that facilitates the processing of irrelevant information, could potentially enhance creative processes.

The results presented in this Research Topic are interesting and promising — yet we have to acknowledge that we are just taking the first steps into this field. Future studies should try to collect further and stronger evidence by using strict research protocols, validated assessment tools and larger samples. Evidence should also be confirmed and validated by meta-analytic evidence.

## Author Contributions

All authors listed have made a substantial, direct, and intellectual contribution to the work and approved it for publication.

## Conflict of Interest

The authors declare that the research was conducted in the absence of any commercial or financial relationships that could be construed as a potential conflict of interest.

## Publisher's Note

All claims expressed in this article are solely those of the authors and do not necessarily represent those of their affiliated organizations, or those of the publisher, the editors and the reviewers. Any product that may be evaluated in this article, or claim that may be made by its manufacturer, is not guaranteed or endorsed by the publisher.
